# Long-Read Sequencing and Structural Variant Detection: Unlocking the Hidden Genome in Rare Genetic Disorders

**DOI:** 10.3390/diagnostics15141803

**Published:** 2025-07-17

**Authors:** Efthalia Moustakli, Panagiotis Christopoulos, Anastasios Potiris, Athanasios Zikopoulos, Despoina Mavrogianni, Grigorios Karampas, Nikolaos Kathopoulis, Ismini Anagnostaki, Ekaterini Domali, Alexandros T. Tzallas, Peter Drakakis, Sofoklis Stavros

**Affiliations:** 1 Laboratory of Medical Genetics, Faculty of Medicine, School of Health Sciences, University of Ioannina, 451 10 Ioannina, Greece; thaleia.moustakli@gmail.com; 2 Second Department of Obstetrics and Gynecology, University Hospital “Aretaieion”, Medical School, National and Kapodistrian University of Athens, 115 28 Athens, Greece; panchrist@med.uoa.gr (P.C.); karampasgr@yahoo.gr (G.K.); 3 Third Department of Obstetrics and Gynecology, University General Hospital “ATTIKON”, Medical School, National and Kapodistrian University of Athens, 124 62 Athens, Greece; thanzik92@gmail.com (A.Z.); isanagnostaki3@gmail.com (I.A.); pdrakakis@med.uoa.gr (P.D.); sfstavrou@med.uoa.gr (S.S.); 4 First Department of Obstetrics and Gynecology, Alexandra Hospital, Medical School, National and Kapodistrian University of Athens, 115 28 Athens, Greece; dmavrogianni@med.uoa.gr (D.M.); nickatho@gmail.com (N.K.); kdomali@yahoo.fr (E.D.); 5 Department of Informatics and Telecommunications, University of Ioannina, Kostakioi, 471 50 Arta, Greece; tzallas@uoi.gr

**Keywords:** genome sequencing, genetic diagnosis, clinical genomics, structural variants, copy number variations, bioinformatics, machine learning

## Abstract

Rare genetic diseases are often caused by structural variants (SVs), such as insertions, deletions, duplications, inversions, and complex rearrangements. However, due to the technical limitations of short-read sequencing, these variants remain underdiagnosed. Long-read sequencing technologies, including Oxford Nanopore and Pacific Biosciences high-fidelity (HiFi), have recently advanced to the point that they can accurately find SVs throughout the genome, including in previously unreachable areas like repetitive sequences and segmental duplications. This study underscores the transformative role of long-read sequencing in diagnosing rare diseases, emphasizing the bioinformatics tools designed for detecting and interpreting structural variants (SVs). Comprehensive methods are reviewed, including methylation profiling, RNA-seq, phasing analysis, and long-read sequencing. The effectiveness and applications of well-known tools like Sniffles2, SVIM, and cuteSV are also assessed. Case studies illustrate how this technique has revealed new pathogenic pathways and solved cases that were previously undetected. Along with outlining potential future paths like telomere-to-telomere assemblies and pan-genome integration, we also address existing issues, including cost, clinical validation, and computational complexity. For uncommon genetic illnesses, long-read sequencing has the potential to completely change the molecular diagnostic picture as it approaches clinical adoption.

## 1. Introduction

Rare genetic disorders are uncommon but collectively affect around 300 million people worldwide. These conditions are typically long-lasting, progressive, and potentially life-threatening, with many patients facing an extended and often years-long search for a definitive diagnosis [1]. The genetic variability that characterizes uncommon diseases is one of the most significant barriers to obtaining fast and accurate diagnoses. Despite significant advancements in next-generation sequencing (NGS)—particularly whole-exome and whole-genome sequencing—that have greatly improved the detection of single-nucleotide variations (SNVs) and small insertions or deletions (indels), many cases remain unsolved [2,3]. This demonstrates that some disease-causing variations may be beyond the detection capability of current short-read sequencing tools [4].

These hard-to-detect variants consist of SVs, a diverse group of genomic changes including translocations, inversions, deletions, duplications, insertions, and more intricate rearrangements, usually spanning 50 base pairs (bp) or more [5]. Through coding sequence disruption, gene dosage changes, or regulatory element perturbations, SVs can significantly impact genome function. Emerging evidence indicates that SVs account for a significant proportion of pathogenic variants in undiagnosed rare genetic diseases [6]. However, detecting SVs remains a formidable challenge, especially when they are located within repetitive or low-complexity genome regions, which are poorly resolved by short-read sequencing [7].

Conventional methods for detecting SVs, including chromosomal microarrays, karyotyping, and targeted PCR-based assays, offer inadequate resolution and efficiency required for comprehensive genome-wide analysis [8]. Although short-read sequencing is effective, it has basic limitations in detecting large or complex structural variants due to its use of reads limited to 150 to 300 base pairs. Undetected or mischaracterized structural changes can lead to missed diagnoses and a poor knowledge of the genetic basis of rare diseases [9,10].

Long-read sequencing has emerged as a potent and transformational technique for detecting and analyzing structural variations. PacBio and Oxford Nanopore (ONT) sequencing methods yield long reads ranging from several kilobases to more than a megabase, allowing for a more contiguous and thorough genome overview. These methods allow for more precise and reliable detection of SVs, especially in genomic regions that short-read methods cannot access [11]. Long-read sequencing technology has advanced the detection of complex structural variations, boosted the accuracy of allele phasing, and uncovered genetic variations that were previously missed in patients with rare disorders [12].

This review underscores the value of long-read sequencing in identifying structural variations and advancing the diagnosis of rare genetic diseases. Long-read technologies deepen our insight into genomic variation by exposing regions that were previously hidden or not well understood, thus supporting the progress of precision diagnostics. We review current methods, advancements in emerging technologies, clinical integration, and the future outlook for incorporating long-read sequencing into mainstream diagnostic workflows.

## 2. Technological Landscape

Long-read sequencing technologies have significantly increased the ability of genomics research and clinical diagnostics to investigate previously poorly characterized genomic regions. PacBio HiFi sequencing and ONT have risen as the leading platforms, presenting unique strengths and compromises across read length, accuracy, throughput, and cost [10,13].

### 2.1. PacBio HiFi Sequencing

PacBio’s HiFi sequencing uses circular consensus sequencing (CCS), which involves repeatedly sequencing individual DNA molecules to obtain a precise consensus read. HiFi reads generally range from 10 to 25 kilobases (kb) and achieve base-level accuracy exceeding 99.9% (Q30–Q40). Owing to its high fidelity, HiFi sequencing is especially valuable for accurate structural variant detection, comprehensive haplotype phasing, and the differentiation of closely homologous sequences, such as pseudogenes and repetitive elements within the genome [12,14].

PacBio platforms, notably the Sequel IIe system, have significantly enhanced throughput while reducing the cost per sample. Despite this, PacBio sequencing is still very expensive per genome and has somewhat shorter read lengths than ONT. Nonetheless, its high accuracy makes it the ideal choice for clinical-grade applications such as the detection of uncommon genetic disorders, where variant calling precision is critical [15].

### 2.2. Oxford Nanopore Sequencing

Oxford Nanopore Technologies provides a completely different way to sequencing by detecting nucleotide sequences as single DNA or RNA molecules pass through a protein nanopore implanted in a synthetic membrane. This methodology enables the generation of ultra-long reads, with lengths surpassing 1 megabase (Mb), thereby offering unparalleled resolution of large or complex structural variants and repetitive genomic regions [16].

ONT platforms span from portable, low-throughput devices like the MinION to high-throughput systems like the PromethION, meeting a diverse variety of scientific and clinical requirements [16]. Although ONT read accuracy has traditionally lagged behind that of PacBio, recent advancements in basecalling algorithms (such as Bonito and Dorado) and improvements in sequencing chemistry (notably Q20+ chemistry) have elevated accuracy beyond 99%, enhancing its competitiveness for clinical applications [17]. ONT’s scalability, minimal capital investment, and rapid real-time sequencing capabilities make it particularly appealing for point-of-care diagnostics and field-based studies [10].

### 2.3. Fundamental Differences Between PacBio and ONT Sequencing

The PacBio HiFi sequencing method uses circular consensus sequencing (CCS), which reads DNA molecules several times to produce extremely accurate consensus reads that are usually 10–25 kb long and have a base accuracy of greater than 99.9%. In contrast, ONT sequences single DNA or RNA molecules as they pass through a nanopore, producing ultra-long reads often exceeding 1 megabase [12]. Despite ONT’s historical inferiority to PacBio in accuracy, current developments in chemistry and basecalling algorithms have raised accuracy to more than 99%. For applications needing high precision, such as clinical diagnostics, PacBio’s platform is recommended despite being more expensive and producing shorter reads. ONT is ideally suited to field-based or point-of-care applications and the detection of complicated structural variants because it provides increased scalability, mobility, and quick real-time sequencing [18]. 

### 2.4. Comparative Summary

PacBio HiFi and ONT platforms offer beneficial techniques for discovering structural variants in rare genetic conditions. PacBio’s exceptional accuracy is particularly suited to clinical use, whereas ONT’s adaptability and extended read lengths facilitate the analysis of intricate genomic rearrangements [19]. Progressive advances in sequencing technologies have led to an increase in the use of hybrid techniques that leverage each platform’s complementary strengths to improve diagnostic precision and yield [20]. Table 1 summarizes the key features of PacBio HiFi and ONT sequencing platforms.

### 2.5. Benchmarking Performance for Structural Variant Detection

Benchmarking studies have allowed researchers to assess the performance of long-read sequencing technologies in SV identification and rare illness diagnosis [21,22]. Comparative evaluations have consistently demonstrated that PacBio HiFi sequencing and ONT possess distinct strengths, with both platforms achieving substantial performance improvements in recent years driven by advances in chemistry, basecalling algorithms, and computational methodologies [23].

In the context of variant calling accuracy, the PrecisionFDA Truth Challenge V2 provided a comprehensive evaluation of SV detection performance across sequencing technologies [20]. PacBio HiFi consistently delivered top performance in structural variant detection, attaining F1 scores greater than 95%. This high level of precision stems from HiFi reads’ exceptional base-level accuracy (Q30–Q40), which minimizes false positives and enables the confident detection of variants in both unique and repetitive genomic regions [24]. Conversely, ONT displayed higher recall rates for specific classes of SVs, particularly larger or more complex rearrangements; however, early iterations of the technology were limited by higher base error rates, leading to less precision. However, recent advancements, such as the use of Q20+ chemistry and updated basecalling models like Dorado, have decreased the performance difference. ONT’s current sequencing generates SV calling F1 scores ranging from 85 to 90%, depending on the genomic context and variant type [14,25].

Read mapping and genome assembly metrics further highlight the complementary advantages of these platforms. According to a study, PacBio HiFi provides extraordinarily high alignment accuracy (>99.8%) and consistent coverage, even in low-complexity regions that are prone to mismapping with conventional technologies [26]. These qualities make it ideal for clinical diagnostics, where reducing false-positive variant calls is critical. ONT’s capability to generate long reads allows for the resolution of large structural variants and repetitive regions that are generally inaccessible using short-read sequencing [27]. This advantage allows ONT to resolve large structural variants and repetitive sequences that are typically inaccessible with shorter read lengths.

Clinical studies further underscore the diagnostic impact of long-read sequencing. Following extensive short-read sequencing without a diagnosis, PacBio HiFi whole-genome sequencing increased diagnostic yield by 10–15% in uncommon illness populations [28]. These cases frequently encompassed cryptic structural variants, phasing-dependent compound heterozygous mutations, or repetitive expansions that eluded detection by conventional methodologies [29]. Similarly, ONT sequencing has been instrumental in uncovering large insertions, tandem repeat expansions, and intricate rearrangements, especially in neurodevelopmental and neurological diseases. ONT sequencing has facilitated the discovery of pathogenic variants in patients with undiagnosed epileptic encephalopathy, muscular dystrophies, and intellectual disabilities that were not detected by chromosomal microarrays and short-read genome sequencing [30].

PacBio HiFi presently incurs higher per-sample sequencing costs, covering both library preparation and sequencing reagents [31]. Conversely, ONT provides pricing alternatives that are more flexible and scalable. Whole-genome sequencing with ONT can be significantly more cost-effective, depending on the platform (e.g., MinION vs. PromethION) and the throughput approach used, making it an appealing choice for high-throughput research or clinical screening applications [32].

Collectively, these benchmarking studies lay the groundwork for selecting an effective long-read sequencing technology depending on the specific needs of a study or clinical use case [33]. ONT offers unparalleled read lengths, cost-effectiveness, and scalability, making it ideal for exploratory research and the investigation of large or complex genomic rearrangements. In contrast, PacBio HiFi provides remarkable precision, making it especially well-suited to clinical diagnostics. As technology continues to advance, hybrid methods that combine the precision of HiFi sequencing with the long-read capabilities of ONT are anticipated to play a key role in the discovery of structural variants [16]. Table 2 compares PacBio HiFi and ONT (Q20+/R10.4.1) sequencing platforms across key metrics for structural variant analysis.

## 3. Structural Variant Detection

SVs are genomic modifications that affect vast regions of DNA, often 50 bp or more. They cover a wide spectrum of variation types, including deletions, duplications, insertions, inversions, translocations, and more complex rearrangements [34]. SVs can disrupt gene coding sequences, change gene dosage, relocate regulatory elements, and modify chromatin architecture, all of which can have a significant impact on gene expression and phenotype. SVs are increasingly recognized as significant contributors to the genomic architecture of uncommon illnesses, cancer, and neurodevelopmental disorders [35,36].

Several repeating elements, such as Alu repeats and retrotransposons, are among the many SVs found in every human genome. Although improved datasets have improved the ability of analytical methods to filter and interpret these variants, difficulties still exist because of the size and complexity of the SVs that are present.

Despite their clinical relevance, accurately detecting structural variants remains a major challenge within the field of genomics. Short-read sequencing, which typically produces reads of 150 to 300 base pairs, is inherently limited in its ability to detect structural variations [37]. Short reads, for example, frequently do not cover the entire length of larger variants, making precise breakpoint resolution difficult. Second, relying on reference genome alignment hinders accurate mapping in repetitive, low-complexity, structurally polymorphic regions, which are particularly rich in structural variants. Third, many structural variants, particularly insertions and complex rearrangements, may be absent or underrepresented in the reference genome, resulting in their omission or inaccurate identification [38].

Long-read sequencing, however, detects SVs significantly more frequently. Long-read sequencing can detect up to 20,000–30,000 SVs per genome, according to recent research. This is up to three–six times more sensitive than short-read sequencing, and even ten times more for insertions and complicated rearrangements in repetitive regions like retrotransposons and Alu elements [39].

Long-read sequencing technologies from PacBio and Oxford Nanopore have substantially enhanced structural variant detection capabilities by producing reads that span from several kilobases up to megabase lengths [7,40]. Long reads enable direct coverage of structural variant breakpoints, improving both the accuracy and comprehensiveness of variant detection. Additionally, this sequencing approach supports de novo assembly and precise haplotype phasing, which are essential for resolving compound heterozygosity and complex allelic variants in rare genetic disorders. Furthermore, by collecting previously inaccessible genomic regions, long-read approaches lessen reliance on reference-based alignment, revealing unique or complex SVs that short-read sequencing would not discover [41].

To harness the power of long-read sequencing for SV detection, a number of specialized computational tools have been developed. Sniffles2, SVIM, PBSV, and cuteSV are among the most commonly utilized. These tools are specifically developed to deal with the unique aspects of long-read data, such as increased error rates and varying read lengths [38,41].

Sniffles2, the successor to the widely used Sniffles program, allows for fast and sensitive SV calling over a wide range of variation types and is compatible with several systems, including PacBio HiFi and ONT [41]. The structural variant identification method (SVIM) is optimized for ONT data and employs a probabilistic model to identify SVs with great sensitivity, particularly in repeated regions. PacBio developed PBSV for HiFi readings, which provides great precision in clinical environments where accuracy is crucial [42,43]. A prominent example of modern structural variant detection tools, cuteSV offers robust compatibility with Oxford Nanopore and PacBio data [44]. Its rising prominence in genomics research is due to its efficient performance, scalability, and user-friendly interface, making it well-suited to large-scale variant analysis.

However, these tools also exhibit certain limitations. Due to alignment ambiguities, Sniffles2, despite its excellent sensitivity, may produce false positives in repeating sections. When SVIM detects minor insertions and deletions, its precision may be diminished [45]. Despite being tailored for PacBio HiFi data, PBSV is not very useful for ONT datasets. Similarly, compared to assembly-based methods, cuteSV’s computational efficiency comes at the expense of a marginally reduced sensitivity for intricate rearrangements. Addressing these restrictions is crucial when evaluating SV calls in research or clinical settings [46].

Collectively, these tools facilitate the effective utilization of long-read sequencing data by researchers and clinicians for accurate and comprehensive SV detection [47]. Continued advances in computational approaches and sequencing technologies will make routine resolution of complicated genomic rearrangements feasible, bringing us closer to a thorough understanding of the genetic causes of rare diseases [48].

## 4. Applications in Rare Disease Diagnostics

Rare genetic disorders, which collectively affect millions worldwide, often involve complex genomic alterations that evade detection by conventional sequencing techniques [49]. While short-read exome and genome sequencing have revolutionized the diagnostic landscape over the past decade, they still leave a substantial proportion of cases—often 40–60%—without a molecular diagnosis. Many of these unsolved cases are now believed to result from SVs, repeat expansions, and other non-coding or complex mutations that fall beyond the technical reach of short-read platforms [50]. Long-read sequencing technologies are increasingly being applied to fill this diagnostic gap, with growing success in uncovering elusive genetic variants that are causally linked to rare diseases [51].

Long-read sequencing excels at resolving pathogenic repeat expansions, a class of genetic variations associated with a broad spectrum of neurological and neuromuscular disorders [52]. Short tandem repeat (STR) expansions, often occurring in intronic or untranslated regions, are the genetic basis of conditions such as Friedreich’s ataxia, myotonic dystrophy, fragile X syndrome, and several types of spinocerebellar ataxia [53]. While long-read platforms can directly sequence through enlarged alleles and precisely quantify repeat size and motif structure, traditional short-read sequencing usually falls short in characterizing the complete length and structure of these repeats. For example, Oxford Nanopore sequencing has been successfully used to detect pathogenic GAA expansions in the FXN gene in Friedreich’s ataxia, while PacBio HiFi sequencing has shown high concordance with gold-standard methods in sizing CTG repeats in myotonic dystrophy type 1 [11].

Beyond the identification of repeat expansions, long-read sequencing approaches have uncovered a wide spectrum of structural variants that have historically evaded detection by conventional short-read whole-exome (WES) and whole-genome sequencing (WGS) technologies [54]. A notable investigation by the Rare Genomes Project underscored the value of PacBio HiFi sequencing in individuals with rare disorders who had previously received comprehensive genetic testing without definitive diagnoses [28]. HiFi sequencing has detected pathogenic insertions, inversions, and complex structural rearrangements across several cases. Examples include a tandem duplication that interfered with a gene essential to neurodevelopment and a deep intronic insertion that produced a unique splicing site that led to abnormal gene expression [55].

Similarly, ONT has been used to detect mosaic chromosomal rearrangements and mobile element insertions responsible for phenotypes such as epileptic encephalopathy, hemophilia A, and congenital malformations [56]. In comparison to conventional exome and genome sequencing techniques, comparative studies demonstrate the complementary nature of long-read sequencing. While WES remains cost-effective and efficient for detecting small coding variants, it misses noncoding regions and has limited utility in SV detection. Large insertion detection, complex structural variant resolution, and phasing issues still plague short-read whole-genome sequencing, despite its improved coverage. Alternatively, long-read whole-genome sequencing delivers a more comprehensive and precise analysis of the genome, enabling improved identification of compound heterozygosity, repeat instability, and structural variants [57,58].

Incorporating long-read sequencing as a second-tier test to several diagnostic pipelines has resulted in a notable improvement in diagnostic yield, usually between 10% and 20%, particularly for patients whose findings from conventional testing were negative [59]. As sequencing costs decrease and analytical methods improve, long-read technologies are anticipated to be increasingly adoption in clinical genomics. These techniques contribute to more precise diagnoses and improved genotype–phenotype correlations through enhanced insight into disease mechanisms and more effective identification of pathogenic variants, thereby supporting more informed clinical decision-making for patients with rare genetic disorders [60]. Table 3 summarizes representative examples of rare disease groups where long-read sequencing has enabled the detection of pathogenic structural variants, including repeat expansions, deep intronic insertions, and complex rearrangements.

## 5. Challenges and Limitations

Although there are currently several barriers preventing long-read sequencing from being widely used in clinical and research settings, it has the potential to completely transform the diagnosis of rare diseases. These limitations encompass various sectors, including financial, computational, and regulatory areas, each of which poses specific challenges to routine implementation [60].

The most notable obstacles are accessibility and expense. On a per-sample basis, long-read sequencing is still far more expensive than short-read sequencing, despite recent dramatic cost reductions. Whole-genome long-read sequencing remains costly (USD 500 to USD 1500) per sample, depending on the platform and throughput [61,62]. Additional expenses may be incurred for data processing, instrument operation, and library preparation. This cost remains prohibitive for many healthcare systems, particularly in low- and middle-income countries. Furthermore, not all laboratories have access to long-read sequencing systems such as Oxford Nanopore PromethION or PacBio Sequel IIe, which limits their viability for use in smaller or resource-constrained labs [63].

Beyond the cost of sequencing, a significant bottleneck is posed by computational and bioinformatics challenges. Large and intricate, long-read datasets necessitate a strong infrastructure for processing, storing, and analyzing data. There is no general gold-standard pipeline, although new structural variant calling tools like Sniffles2, SVIM, PBSV, and cuteSV are tailored for long-read data. Performance can vary depending on platform, variant class, and genome context. Furthermore, long-read data, especially from ONT, can still contain residual base-level errors that complicate small variant calling or interpretation in coding regions. The integration of multiple variant types (e.g., SNPs, SVs, repeat expansions) into a single unified analysis remains technically demanding and often requires expertise that is not readily available in all clinical laboratories [21,64].

A third major challenge lies in the absence of standardized protocols, validation frameworks, and effective clinical integration. Regulatory agencies have started to recognize the benefits of long-read technologies; however, clinical implementation guidelines are still under development. The wide range of variant types and the rapid evolution of sequencing platforms present significant challenges in thoroughly establishing analytical validity, reproducibility, and clinical relevance when validating assays for diagnostic use [65]. In addition, there is a pressing demand for benchmarking datasets, standardized reference materials, and best practice protocols for the detection and interpretation of SVs. The lack of standardized protocols complicates the comparison of results between laboratories and hinders adherence to regulatory and accreditation standards [66].

Larger SVs, particularly those in noncoding areas, are challenging to report in a clinical setting because the majority of reporting recommendations, including ACMG standards, are currently made for single nucleotide variations (SNVs) and small indels [67]. Improvements to these frameworks will be necessary to integrate long-read SV data, such as more precise classification criteria for the pathogenicity of SVs and functional validation techniques for complicated and noncoding SVs. To establish strong guidelines for SV interpretation and reporting, cooperation between sequencing consortia, clinical geneticists, and regulatory agencies is essential [68].

Interpreting structural variations clinically is another difficult challenge. While structural variant detection techniques have improved, accurately determining pathogenicity continues to be a significant challenge, especially for complex or noncoding variants. Variants of unknown significance (VUS) account for a large number of discovered SVs, and interpretation is further constrained by the absence of extensive demographic and disease-specific databases [69]. Efforts such as the Genome in a Bottle Consortium and initiatives to expand SV annotations in gnomAD are steps in the right direction, but broader adoption of long-read data in clinical variant databases is still needed [70].

**Figure 1:**
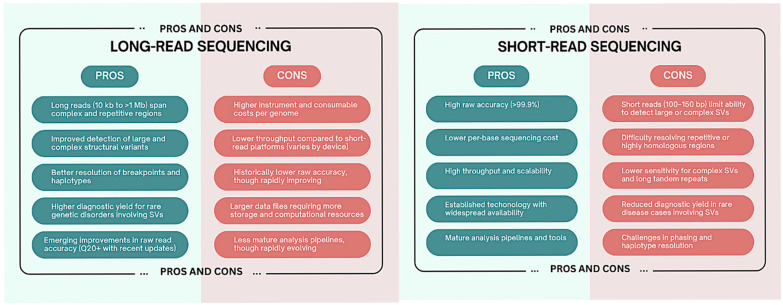
This figure compares the strengths and limitations of short-read and long-read sequencing technologies in the context of structural variant detection and rare disease diagnostics.

Long-read sequencing technologies have a lot to offer in the diagnosis of rare diseases, but they will need to overcome obstacles in the areas of cost, computational complexity, clinical interpretation, and analytical standardization before they can be fully integrated into clinical genomics. For long-read sequencing to fully realize its potential in precision medicine, these challenges must be addressed by cooperative benchmarking, infrastructure investment, and regulatory framework development [11]. Figure 1 summarizes the strengths and limitations of short-read and long-read sequencing technologies in the context of structural variant detection and rare disease diagnostics.

## 6. Future Directions

The landscape of rare disease research is set to undergo significant transformation with the ongoing advancements in long-read sequencing technologies and their expanding integration into routine genomics and clinical diagnostic workflows. Ongoing advancements are expected to enhance the scope, accuracy, and accessibility of structural variant detection at both individual and population scales, while addressing current limitations [71].

An effective approach involves the application of hybrid sequencing techniques, which leverage the long-range continuity provided by long-read platforms in conjunction with the high base-level accuracy characteristic of short-read sequencing [19]. This combined strategy has already proven beneficial in decreasing overall costs and improving both the sensitivity and phasing accuracy of variant detection. Hybrid assemblies provide a more thorough and nuanced view of individual genomes by enabling minor variation and SV detection in research and diagnostic pipelines [72].

For instance, complex genomic rearrangements, insertion–deletion events, and cases of compound heterozygosity, often missed when using either sequencing method in isolation, can be reliably resolved with high confidence by integrating Illumina short reads with PacBio HiFi or ONT long reads [73].

The use of targeted capture and enrichment technologies to improve long-read sequencing efficiency is also gaining attention. Selective sequencing of therapeutically relevant regions can be achieved using methods including hybridization-based capture, adaptive sampling (on ONT platforms), and Cas9-mediated enrichment [74]. In diagnostic applications where whole-genome sequencing may not be required, these techniques are particularly helpful since they enrich for target loci, increasing coverage depth, decreasing data complexity, and lowering sequencing costs [75].

Another groundbreaking advancement is the implementation of graph-based reference genomes, which move beyond the limitations imposed by a single linear reference. Conventional genome alignment maps sequencing reads to a standardized reference genome, a method that frequently fails to capture highly variable regions and genetic variation across populations [11]. Graph genomes improve read alignment, minimize reference bias, and increase the accuracy of variant detection, especially for structural variants and population-specific alleles, by incorporating multiple haplotypes and structural arrangements into a single comprehensive framework [76].

Rare diseases can be influenced by population-specific variants. With the increasing availability of long-read sequencing data, employing graph-based reference genomes will enhance both the accuracy and inclusivity of genomic analyses across diverse ancestral groups [77].

The creation of population-scale SV databases, which are essential for distinguishing between benign and pathogenic variations, is occurring concurrently with these technical advancements [78]. High-resolution catalogs of SVs across diverse populations are being generated through initiatives such as the Human Pangenome Reference Consortium, alongside ongoing efforts to integrate long-read SV calls into comprehensive resources like gnomAD. These databases will greatly increase the precision and effectiveness of variant interpretation by offering allele frequency data, enhancing the annotation of noncoding SVs, and lowering the number of variations of ambiguous relevance in clinical workflows [79]. In the long term, the role of structural variants in complex traits, variation penetrance, and genotype–phenotype correlations will be more thoroughly understood [80].

In the near term, long-read sequencing will likely continue to augment existing short-read approaches rather than entirely supplant them. Clinical labs are anticipated to use long-read technology as a first-line diagnostic assay for genetically undetected patients, rather than merely as a secondary tool, as sequencing costs continue to drop, throughput rises, and automated bioinformatics pipelines become more reliable [81].

In the short future, though, long-read sequencing is probably going to continue to supplement existing short-read methods rather than completely replace them. When routine testing yields equivocal results but there is a high diagnostic suspicion of a genetic condition, its usage may be prioritized. In these situations, long-read platforms are particularly well-suited to phasing variations without parental samples, resolving complex structural variants, and detecting complex genomic rearrangements, exon inversions, or retrotransposed insertions that short-read technologies cannot detect. Targeted application in such challenging contexts represents a pragmatic integration strategy; however, widespread adoption remains limited due to escalating costs and heightened infrastructure demands.

Clinical norms and expectations are changing as early adopters have already started to show the benefits of long-read sequencing in detecting diagnostic variations that traditional approaches miss. To fully exploit the clinical relevance of long-read data, integration with clinical decision support systems, electronic health records, and regulatory frameworks will be necessary [11].

Ultimately, the convergence of long-read sequencing, advanced informatics, and collaborative data sharing promises a future in which comprehensive SV detection is routine, enabling more accurate diagnoses, earlier interventions, and more precise therapeutic strategies for individuals with rare genetic disorders [65].

## 7. Conclusions

The introduction of long-read sequencing has enhanced genomics, providing high resolution for detecting SVs. Platforms such as PacBio HiFi and ONT allow precise detection of insertions, deletions, inversions, repeat expansions, and complex rearrangements related to an array of rare genetic disorders [82]. Its therapeutic value lies in solving unexplained cases, uncovering novel disease processes, and improving genotype–phenotype understanding. By characterizing pathogenic repeat expansions and revealing noncoding SVs, long-read sequencing advances diagnosis and offers hope to affected families [82].

However, wider clinical integration requires reducing costs, improving bioinformatics, establishing reliable pipelines, and standardizing interpretations. Developing graph-based reference genomes, hybrid sequencing approaches, and extensive population-scale SV databases will be essential for promoting widespread adoption [81]. As technology advances and challenges to adoption are overcome, its incorporation into mainstream clinical practice holds enormous promise for providing more accurate, rapid, and thorough diagnoses to patients around the world.

## Figures and Tables

**Table 1:** Key features of PacBio HiFi and ONT sequencing platforms, comparing read length, accuracy, throughput, and costs. Also highlighted are each platform’s unique strengths, such as HiFi’s exceptional accuracy and ONT’s ultra-long reads and portability.

Feature	PacBio HiFi	Oxford Nanopore (ONT)
Read Length	10–25 kb (HiFi reads)	Up to >1 Mb (typical reads 20–100 kb)
Accuracy	>99.9% (HiFi consensus)	~98–99.5% (Q20+ with recent improvements)
Throughput	Moderate–High (up to ~160 Gb/run Sequel IIe)	High (varies by device; PromethION > Tb)
Instrument Cost	High (Sequel IIe system)	Lower (MinION, GridION, scalable options)
Consumable Cost	Higher per Gb	Lower per Gb
Notable Strengths	Exceptional accuracy, suited to clinical applications	Ultra-long reads, portability, real-time analysis

**Table 2:** Comparison of PacBio HiFi and ONT (Q20+/R10.4.1) sequencing platforms across key metrics for structural variant analysis, including accuracy, assembly quality, cost, and diagnostic effectiveness. It highlights each platform’s strengths in SV detection and their impact on rare disease diagnosis.

Metric	PacBio HiFi	ONT (Q20+/R10.4.1)
SV Calling F1 Score	>95%	85–90% (improving with basecaller upgrades)
Typical Assembly N50	20–30 Mb (HiFi reads)	>50 Mb (ultra-long reads)
Pathogenic SV Detection	High precision, fewer false positive	Higher sensitivity for large/repetitive SVs
Per-genome Cost (USD)	USD 1000–1500	USD 400–800 (varies by scale and platform)
Diagnostic Yield Gain (rare disease cases)	+10–15% vs. short-read WGS	Similar, with strengths in large SVs and TRs

**Table 3:** Applications of long-read sequencing technologies in rare disease diagnostics by variant type and disease group.

Variant Type	Disease Group	Representative Diseases	Example of Long-Read Utility
Repeat Expansion	Neurological and Neuromuscular	Fragile X syndrome (FMR1) Friedreich’s ataxia (FXN) Huntington’s disease (HTT) Myotonic dystrophy (DMPK)	Direct sizing of expanded repeats Detection of complex repeat motifs
Deep Intronic Insertion	Neurodevelopmental Disorders	Duchenne muscular dys-trophy (DMD Deep intronic variant) Neurofibromatosis type 1 (NF1)	Identification of pathogenic insertions creating cryptic splice sites
Large Deletions/Duplications	Syndromic Disorders	DiGeorge syndrome (22q11.2 deletion) Charcot-Marie-Tooth dis-ease type 1A (PMP22 du-plication)	Resolution of breakpoints in large CNVs missed by short reads
Mobile Element Insertions	Neurological Disorders	Hemophilia A (F8 intronic insertion) Epileptic encephalopathy (SCN1A insertion)	Detection of Alu, LINE-1 SVA insertions disrupting gene function
Complex Structural Rearrangements	Developmental Disorders	Congenital aniridia (PAX6) Chromothripsis in congen-ital malformations	Phasing and de novo as-sembly to resolve complex rearrangements
Mosaic Structural Variants	Somatic Mosaic Disorders	Pallister-Killian syndrome (isochromosome 12p) Mosaic Turner syndrome	High sensitivity for mosaic SV detection in heteroge-neous samples

## Data Availability

No new data were created or analyzed in this study. Data sharing is not applicable to this article.
